# The structure of extremely long mouthparts in the aphid genus *Stomaphis* Walker (Hemiptera: Sternorrhyncha: Aphididae)

**DOI:** 10.1007/s00435-015-0266-7

**Published:** 2015-04-08

**Authors:** Jolanta Brożek, Ewa Mróz, Dominika Wylężek, Łukasz Depa, Piotr Węgierek

**Affiliations:** Department of Zoology, Faculty of Biology and Environmental Protection, University of Silesia, Bankowa 9, 40-007 Katowice, Poland

**Keywords:** Labium, Maxillae, Mandibles, Morphology, Feeding

## Abstract

Scanning electron microscopy and light microscopy were used to elucidate the morphology of labium and mandibular and maxillary stylets of the aphids *Stomaphis quercus* (L.) and *S. graffii* Cholodkovsky. The mechanism of labium shortening associated with feeding process was described as well. *Stomaphis quercu*s and *S. graffii* have cone-shaped labium of 13 and 10 mm in length, respectively, that strongly extend behind the abdomen. The stylets bundle comprises a pair of mandibular and maxillary stylets which are on average as long as labium. Serial cross sections of labium revealed that the first segment is inverted inside and the second is pulled into it; both segments are shifted into abdomen. This study provides new information on *S. quercus* and *S. graffii* mouthparts that may help to understand their feeding behavior.

## Introduction

One of the most striking characters associated with the Hemiptera is a set of modifications in their mouthparts (labium, labrum, maxillae and mandibles), which are sometimes described as beak like. Usually the labium and rarely the labrum are modified into a rostrum where the mandibles and maxillae form needle-like or thread-like stylets lying within a grooved labium (Capinera 2008).

The mouthparts of aphids (Hemiptera: Aphididae) have been examined in detail by several authors because the insects are important virus vectors. Thin and elongated mouthpart stylets enable aphids to penetrate plant tissue compartments (Powell et al. 2006). Similar to other hemipterans, a stylets bundle consists of a pair of outer mandibular and inner maxillary stylets (Forbes 1977). Both pairs of stylets are involved in the process of tissue penetrating. Mandibles are important in the physical penetration of plant cell walls, but it is the maxillae that perform a major role in host plant selection (Powell et al. 2006). Owing to the microstructure of maxillary stylets, individual plant cells may be penetrated including injection of saliva and uptake of plant sap (Martin et al. 1997; Powell 2005; Prado and Tjallingii 1994). A stylet penetration process enables aphids to puncture the symplast and exploit intracellular compartments without wounding. This behavior is vital for phloem-exploiting insects and helps them to inoculate viruses into vascular and non-vascular plant cells. These subtle interactions with the symplast may be crucial factors for host selection in aphids (Powell et al. 2005).

In the recent ultrastructure study of aphid mouthparts, Uzest et al. (2010) reported the existence of a distinct anatomical structure called the “acrostyle” on the tips of aphid maxillary stylets. It is an expanded part of the cuticle visible in the common duct of all aphid species observed.

Morphological structures of labium in the individual representatives of aphids have been examined and described in many studies. According to Guyton (1924), in the Aphididae, the rostrum (labium) consists of four or five segments, and this segmentation is characteristic of the family. However, a five segmented labium does not occur in other groups of the Hemiptera. The presence of four segments of labium was confirmed in the representatives of Aphidinae, e.g., in *Aphis fabae* by Weber (1928), in *Myzus persicae* by Forbes (1969), *Schizaphis graminum* by Saxena and Chada (1971) as well as in *Euceraphis betulae* (Calaphidinae) by Wojciechowski (1992). The five segmented labium has been described only in the Lachninae (e.g., *Lachnus roboris* (L.)), where the form has resulted from the secondary division of the apical segment (Wojciechowski 1992). Another example of labium modification was observed in *Aphis citricola* van der Goot (Aphidinae) by Razaq et al. (2000); the author documented only three segments there. The labium can differ in length, and in most species, it reaches the coxa (hip) of the third pair of legs. Only in species that feed on the trunks, branches and roots of trees, is the labium as long as the body (Lachninae, Eriosomatinae).

The genus *Stomaphis* Walker (Lachninae) provides an example of extremely long mouthparts in aphids, where the labium greatly exceeds the length of body—it is about twice as long (Pesson 1951; Szelegiewicz 1978). A very long labium and long stylets are adaptations to probing through a particularly thick bark tissue of trees as these aphids feed on tree trunks (Sorin 1966; Depa 2011; Depa and Mróz 2012; Depa et al. 2013). The latter authors have observed that during feeding, the labium of *Stomaphis* shortens externally so that only distal segments are visible while two very long proximal segments are inserted into the body. It seems to be a very specific adaptation.

The aim of this study was to: (1) provide the description of external morphology and internal characters of the labium as well as the internal structures of the maxillary and mandibular stylets of *Stomaphis* and (2) demonstrate the possibility of shifting the first two segments and placing them within the abdominal cavity.

## Materials and methods

The occurrence of different positions of labial segments was documented in the scanning electron microscopy SEM as well as in the light microscope based on general and histological preparations which indicated the structural details of the cross section of the labial segments and stylets in *Stomaphis*. The studied samples comprised two species: *S. quercus* (L.) and *S. graffii* Cholodkovsky.

### Samples in SEM

This study of labium and stylets in *Stomaphisquercus* was conducted on alcohol material of six adult specimens dehydrated in series of alcohol and dried in air. Additionally, one specimen from 70 % ethanol was dehydrated in series of alcohol and acetone and dried in the critical point (CPD), and one more specimen from 70 % ethanol was chemical dried in the hexamethyldisilazane (HMDS). All specimens were obtained from the collection of the Department of Zoology University of Silesia in Katowice.

The basal part of head with a part of the rostrum or the whole specimens was glued onto a scanning electron microscope stub. The materials used for SEM photographs were gold coated; the photographs were taken with a Hitachi UHR FE-SEM SU 8010 scanning electron microscope, with the samples placed in the high-pressure chamber.

### General preparations for the light microscope

The adult aphids (*S. quercus* and *S. graffii*) and larvae (*S. graffii*) were preserved in 70 % ethanol and later transferred to 20 % KOH where they macerated for about 2–3 weeks. Later, specimens which became fully transparent were washed with distilled water and transferred to 90 % glycerin for mounting. Digital images were obtained with a DN-100 camera installed in a Nikon Eclipse-E600 light microscope.

#### Histological preparations

To produce histological preparations, the paraffin method was applied. The material was treated with Carnoy’s solution (glacial acetic acid + absolute ethanol) for 20–30 min and transferred to 70 % ethanol. Next, insects were dehydrated in ethanol of concentration increasing from 90 %, through 96 %, to 100 %. In order to ensure transparency, specimens were kept in methyl benzoate for 24 h. Then, the material was transferred to benzene, to benzene with paraffin (in proportions 5:1, 3:2, 2:3) and kept in paraffin I (melting point 56 °C) and paraffin II (melting point 60 °C) for the night. Finally, it was immersed in paraffin II. The bars obtained were sectioned into 2–5 μm strips with a Reichert (Reichert Austria) rotary microtome. The strips were then stuck on slides in a 0.5 % gelatin solution at temperature 50–60 °C and dried at 40 °C. At this stage, the preparations were deparaffined in benzene and treated with a series of 100–60 % ethanol solutions, rinsed in distilled water, stained with Ehrlich’s acid hematoxylin, oxidized and differentiated with 0.5 % xylidine ponceau. Next, preparations were treated with a series of 100–60 % ethanol solutions, rinsed twice in benzene and embedded in Canadian balm or Di-N-Botyle Phthalate in Xylene (OPX).

For each species, several complete series of microtome sections and total preparations were produced. Digital images were obtained with a DN-100 camera installed in a Nikon Eclipse-E600 light microscope; the measurements were taken using Lucia net software. Figures were obtained with an Olympus SZX9 stereo microscope.

The following abbreviations of morphological structures, their positions and modifications were applied: abd—abdomen, aIIs—apodema of IIs, cl—clypeus, cr—crumena, br—barbs, dl—dorsal lock, e.mbIs—external membranous wall of Is, es—external surface, fc—food canal, he—head, tr—trichoid sensilla, i.mbIs—inverted external membranous wall of Is, is—inverted external surface, Lb—labium, lg—labial groove, lgb—total body length, lgl—labium length, Lr—labrum, m—muscle, mIs—membrane of Is, mb—membrane, Mds—mandibles (right RMd and left LMd), ml—middle lock, Mxs—maxillae (right RMx and left LMx), nc—nerve canal, rb—reinforcement bar, sc—salivary canal, st—stylets/stylete bundle, vl—ventral lock, th—thorax, Is—first labial segment, IIs—second labial segment, IIIs—third labial segment, IVs—fourth labial segment and Vs—fifth labial segment.

## Results

### Gross morphology of the mouthparts

**Fig. 1:**
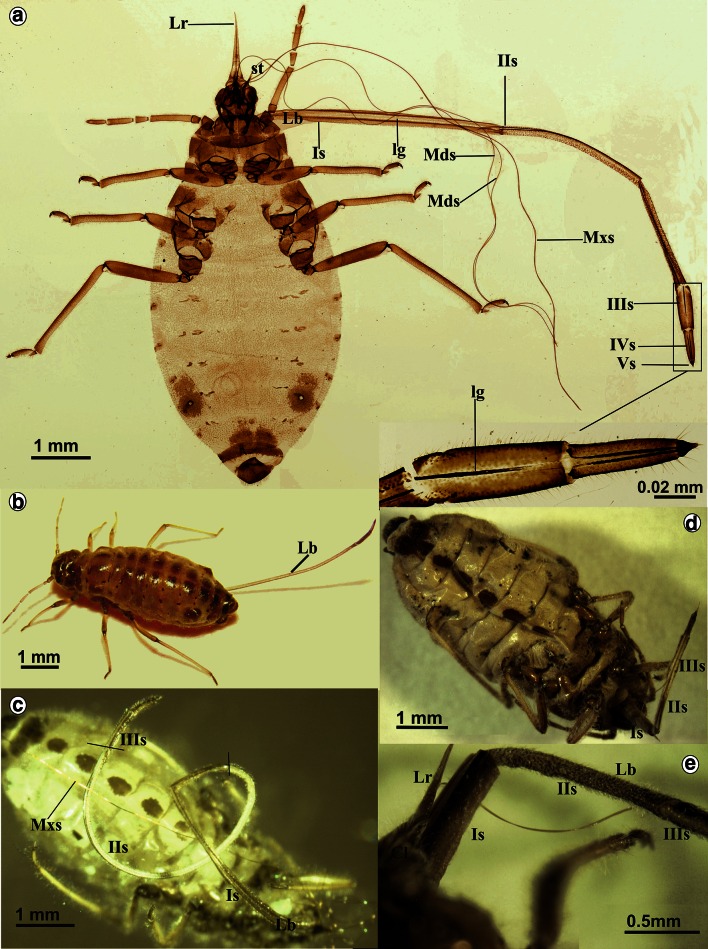
Mouthparts of *Stomaphis graffii*. **a** Shape and length of individual labial segments (I–Vs) in the rest, conical, short labrum (*Lr*), looped the stylets bundle (*st*)—mandibles (*Mds*) and maxillae (*Mxs*), *lg* labial groove in individual segments. **b** Natural position of the labium in living non-feeding aphid. Mouthparts of *Stomaphis quercus*. **c** Length of labium (Is–Vs) at rest. **d**, **e** Shortening the labium during feeding, parts of the first and second segments are visible

The mouthparts of *Stomaphis* externally include the labrum (Lr), labium (Lb) also called rostrum and a stylets bundle (st) (Fig. 1a) consisting of two mandibular (Md) and two maxillary (Mx) stylets. The shape and length of the labium and the stylets in *Stomaphis* were analyzed at two stages, i.e., while resting (when not feeding) (Fig. 1a–c) and during feeding (Fig. 1d, e).

Labrum is short and reaches one-third of the length of the first segment of labium (Fig. 1a) or the proximal part of the fourth segment while feeding. The cone-shaped labrum is attached to the clypeus, and in natural position, it overlies the labial groove (lg) of the labial segments (Fig. 1e).

**Fig. 2:**
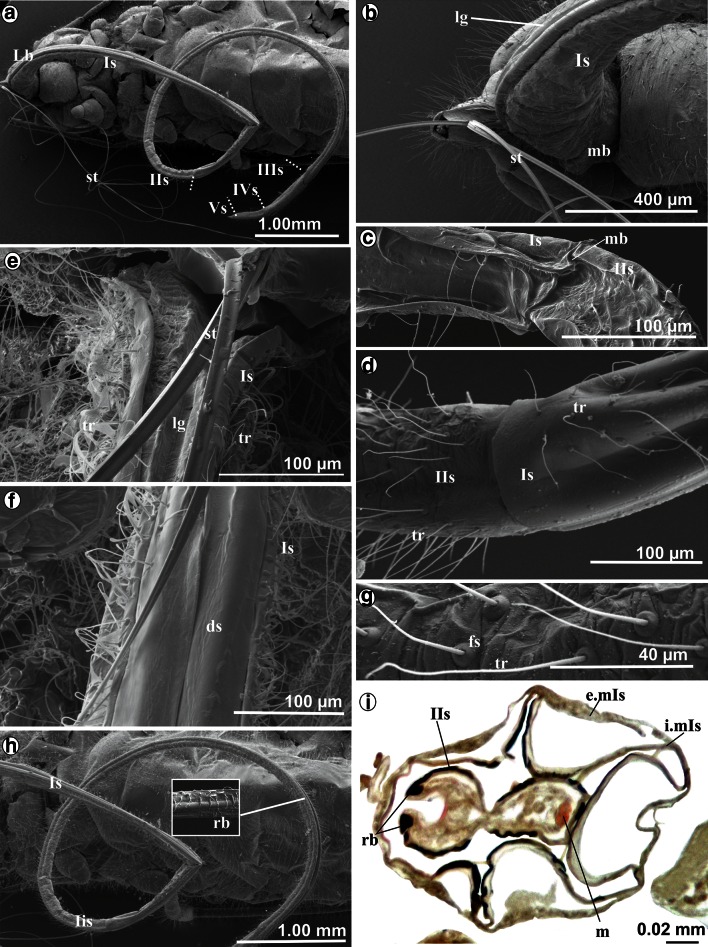
Shape of the labium and length of stylets at rest in *S. quercus*. **a** Length of the labial segments Is–Vs. **b** A wide and more chitinous basal part of the first segment and the membranous connection with the head, **c** mb Membranous connection between the first and second segment, dorsal view. **d** Arrangement of the trichoid sensilla on the first and second segment, lateral view. **e** *lg* Shallow labial groove in the base of first segment. **f** *ds* Dorsal side of the first segment. **g** *tr* Trichoid mechanosensilla inserted in flexible sockets. **h** *rb* Reinforcement bars on the dorsal side of the second segment*. S. graffii*. **i** *rb* Reinforcement bars of the second segment in cross section visible inside the first segment

Labium is very long and exceeds far beyond the body length, i.e., it is more than twice as long (Figs. 1a–c, 2a, Table 1(A)). During resting (when not feeding or after molting), all segments of the labium (Lb) are protruding externally (Figs. 1a, c, 2a) and the labium is pulled by an aphid along the surface of the host plant, usually with its distal end bent over the abdomen (Fig. 1b).

**Table 1:** Length of labial segments at rest (A) and during feeding (B) in *Stomaphis quercus and S. graffii;* measurements based on specimens observed in stereo microscope and on SEM images

Segments of labium	First	Second (µm)	Third (µm)	Fourth	Five (µm)	Total length (µm)
A *Stomaphis quercus*	5700 µm	5600	900	600 µm	190	12,990
B *Stomaphis quercus*	In body	300	900	600 µm	190	1990
A *Stomaphis graffii*	4380 µm	4340	730	540 µm	100	10,090
B *Stomaphis graffii*	In body	250	730	54 µm	100	1620
Stylets at rest	*Maxillae*			*Mandibles*		
*Stomaphis graffii*	10,040 µm			10,040 µm		
*Stomaphis quercus*	11,690 µm			11,690 µm		

The length of the rostrum and stylets is measured by the distance between the base and the apex

Length of the body of *Stomaphis quercus* = 6.45 mm, total length of the labium at rest = 13 mm, length of the labium during feeding ≥2 mm

Length of the body of *Stomaphis graffii* (apterous morph) = 5.85 mm, total length of the labium at rest = 10 mm, length the labium during feeding ≥1.5 mm

The measurements were taken for each species on the base of single specimen from the preparation

Labium consists of five segments (Is, IIs, IIIs, IVs and Vs) (Figs. 1a, c, 2a), each having a labial groove (lg) (Fig. 2b) which encases a stylets bundle.

The *first* segment is suspended ventrally from the neck membrane (mb) of the head (Fig. 2b). The length of this segment is 4380 µm in *S. graffii* and 5700 µm in *S. quercus* (Table 1(A)). The proximal part of this segment is more sclerotized and slightly wider (Fig. 2b), while the distal part is more membranous (Fig. 2c) and shaped as an extremely elongated tube (Fig. 2d). The labial groove is wide and shallow basally and does not hold the stylets strongly (Fig. 2e). The dorsal surface (ds) of the first segment is distinctly different from the lateral side and visible as flexible, longitudinal lobes covering the labial groove (Fig. 2f). This segment is laterally and ventrally covered by smooth, pore less hair-like structures (Fig. 2d, e). These hairs are classified as trichoid sensilla (tr) inserted in the flexible sockets (fs) that perform the mechanosensitive function (Fig. 2d, g) and are clearly present on all segments—they are more numerous on segments II–IV (Fig. 2g) but distinctly fewer on the segment I (Fig. 2d).

The *second* segment is almost the same length (4340 µm) as the *first* one in *S. graffii* (Fig. 1a) and in *S. quercus* (5600 µm) (Fig. 2a) (Table 1A). A membranous (mb) connection between these segments is clearly visible (Fig. 2c). Generally, the *second* segment is also membranous, but it has the chitinous reinforcement bars (rb), which strengthen its dorsal side (Fig. 2h). In this segment, the stylet groove (lg) is more enclosed dorsally and includes the stylets bundle. On the cross section, the reinforcement bars are visible on the dorsal edges of this segment as two dark spots at the labial groove (*S. graffii,* Fig. 2i). On the ventral part of the segment, there are muscles (m) attached (Fig. 2i). The diameter of this segment is smaller than that of the *first one*.

**Fig. 3:**
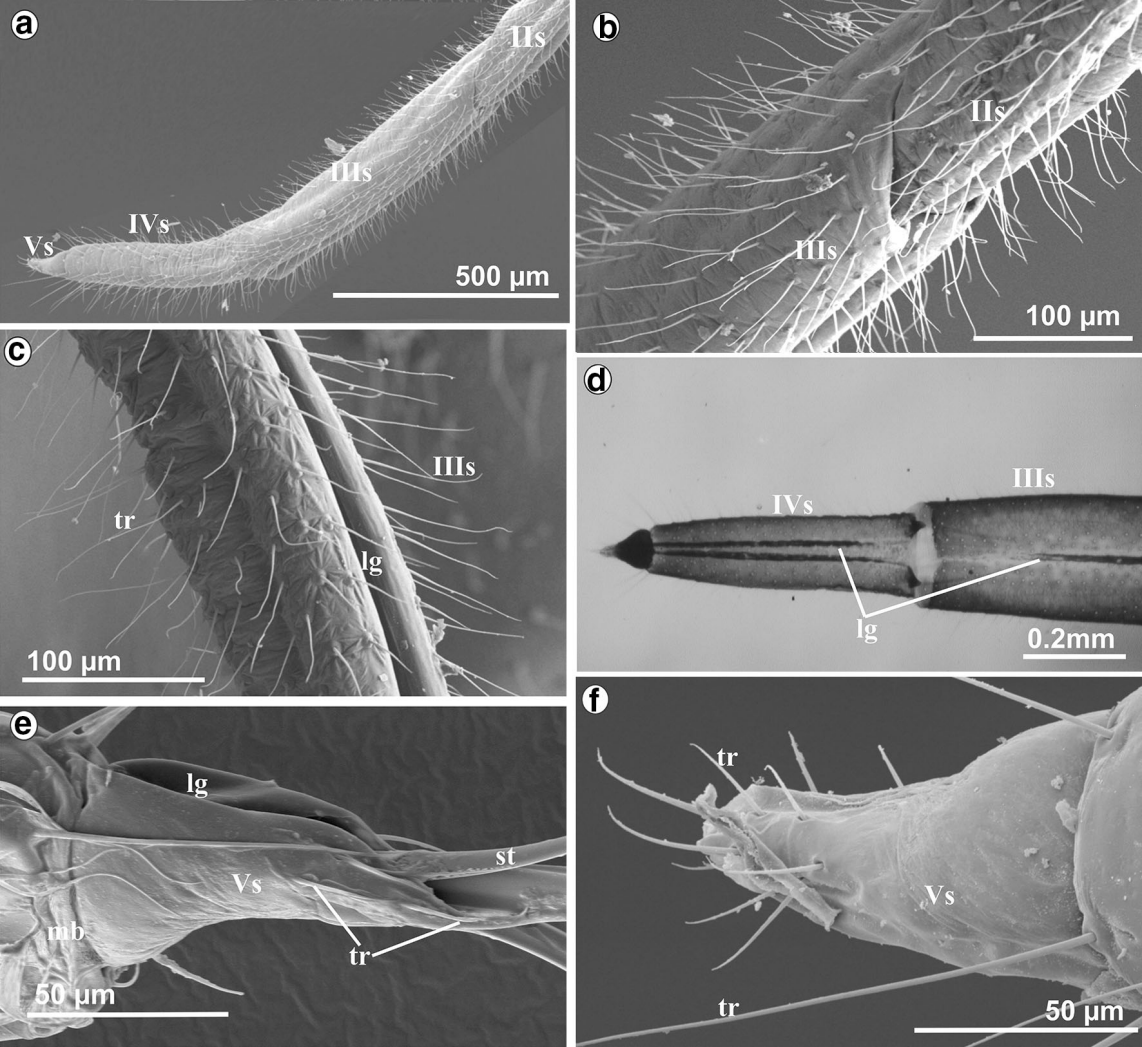
Shape and length of the labium at rest in *S. quercus*. **a** Tubular shape of the segments *IIIs*, *IVs* and *Vs* in magnification. **b** Connection between *III i IV* segments, dorsal view. **c** *lg* Labial groove deeply placed in the third segment which housed the stylet bundle. **d** Labial groove of the third and fourth segment. **e** *lg* Wide labial groove in the fifth segment. **f** *tr* Trichoid mechanosensilla on the ventral side of the fifth segment

In *S. graffii*, *th*e *third* and the *fourth* segments are 730 and 540 µm long, respectively, both are evidently shorter than the *first* and the *second* ones (Table 1A; Fig. 1a). The length of these segments in *S. quercus* is 900 and 600 µm, respectively (Table 1A; Figs. 1c, 3a). The third segment is connected by a membrane with the *second* and *fourth* segments (Figs. 1a, 3a, b). Both segments are tubular in shape, strongly chitinized and the stylet groove is closed, i.e., the dorsal edges are in the direct contact (Figs. 1a, 3c, d).

The distal part of the *fourth* segment is constricted and visibly separated from the *fifth* segment through the membrane (mb) (Fig. 3d–f). The *fifth* segment is the shortest (190 µm in *S. quercus* and 100 µm and *S. graffii*) and constitutes a terminal element of the labium with the evidently wider groove. The stylet end lies in an open labial groove (Fig. 3e). On the labial tip, there are several trichoid sensilla of different length (Fig. 3d–f).

The length of labial segments at rest (A) and during feeding (B) in *Stomaphis quercus and S. graffii;* measurements based on specimens observed in stereo microscope and on SEM images. The length of the rostrum and stylets is measured by the distance between the base and the apex (Table 1).

### Stylets bundle

The right (RMx) and left (LMx) maxillary stylets and right (RMd) and left (LMd) mandibular stylets are like flexible needles, and their length approximates the length of the labium at rest (frequently, on preparations stylets are looped) (Figs. 1a, 2a). The length of mandibular (10,040 µm) and maxillary (10,040 µm) styles is almost the same as labium (10,090 µm) in *S. graffii* and slightly longer in *S. quercus*: Maxillae and mandibles are 11,690 µm long, while labium is 12,990 µm in length (Table 1A).

**Fig. 4:**
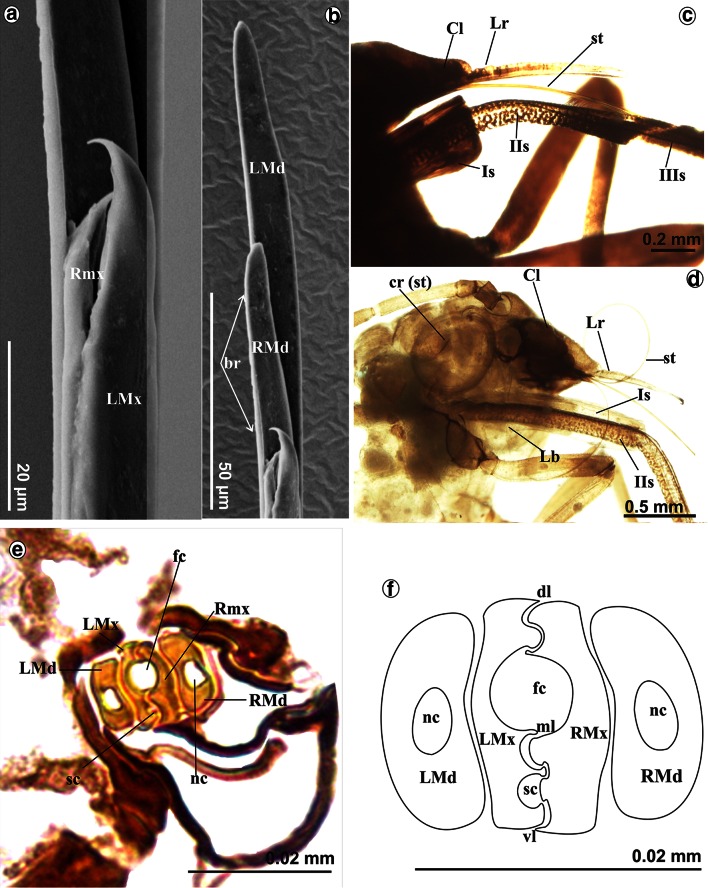
Maxillae and mandibles in *S. quercus*. **a** Pointed tip of the maxillae: *left* (*LMx*) and *right* (*RMx*). **b** Ends of the mandibles with small barbs (*br*) on external side: *left* (*LMd*) and *right* (*RMd*). Evidence of inserting the second segment into the first. **c** The part of the first and second segment visible externally, the stylets bundle come into the groove in the third segment. **d** *st* Stylets rolled up in *cr* crumena inside of the head in the larva (*S. graffii*). Cross section by maxillae and mandibles *S. graffii*. **e** Maxillae (*LMx*, *RMx*) and mandibles (*LMd*, *RMd*) placed in groove of the labium. **f** System connection between the maxillae. *dl* dorsal lock, *ml* middle lock, *vl* ventral lock, *sc* salivary canal, *fc* food canal

The external edges of maxillae are smooth, and the end is pointed and slightly flexed (Fig. 4a), whereas at the end of the mandibles, there are five small barbs (br) (Fig. 4b).

The stylets are pressed by underside of labrum and are inserted into the distal part of the first segment of the labium (rostrum) (Figs. 2b, 4c) and passed to following rostral segments. These stylets emerge from the head into the labial groove, running along the dorsal surface of the labium. In resting, the stylets are placed in labium because they are of the almost the same length.

During feeding, the stylets are located in the last three segments of the labium (Fig. 3c) and they are significantly protruding beyond the labium.

In the larva (Fig. 4d), the stylets are contained in an internal pouch, the crumena (cr) that extends from the head to the thorax.

### Cross section by maxillae and mandibles

In the cross section, the right (RMx) and left (LMd) maxillae have irregular surface and they are internally connected by the system of ridges and grooves (Fig. 4e). Three points of the internal connection between the maxillae have been found, i.e., the dorsal (dl), middle (ml) and ventral (vl) locks (Fig. 4f). The inner sides of the maxillae form a bigger food canal (fc) and a smaller salivary canal (sc). The latter is placed in the left maxilla while the food is placed centrally, i.e., both maxillae have concave surfaces of the same size (Fig. 4f). In the cross section, external surface of maxillae is smooth and slightly convex. The right (RMd) and left (LMd) mandibles are simple structures with few morphological variations; they are convex externally and concave internally. The internal concave region of mandibles has the function of holding the maxillary stylets, so they are positioned laterally to the external surface of the maxillae. Each mandible possesses the nerve canal (nc).

### The shape and size of labium during feeding

**Fig. 5:**
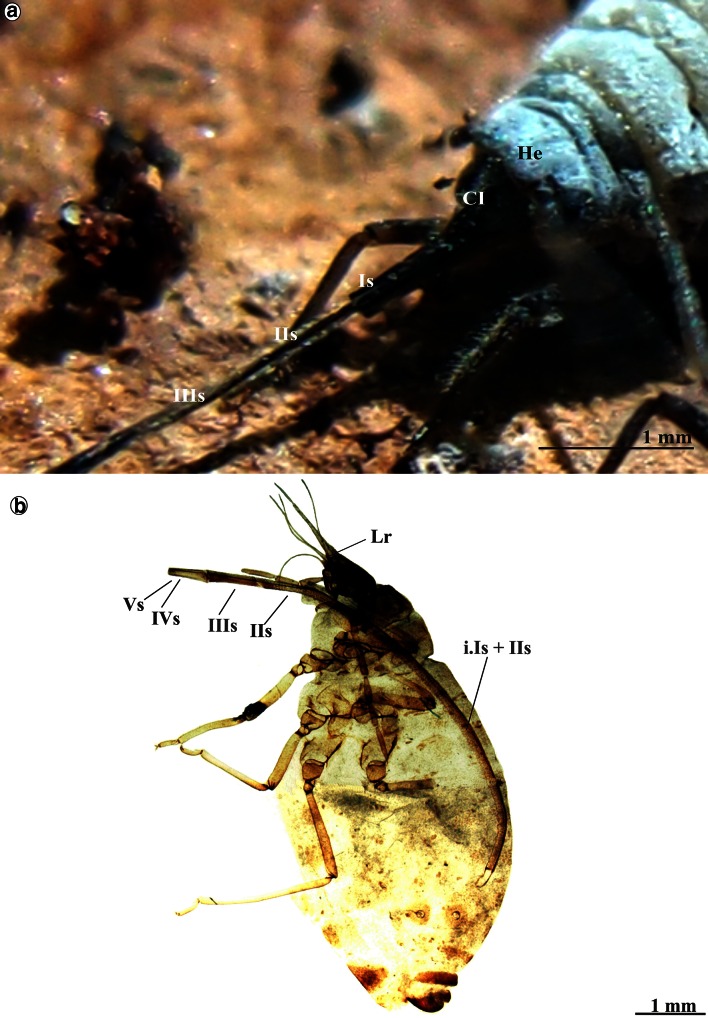
Feeding process in *Stomaphis* *quercus*. **a** Labial segments are situated at some angle to the plant surface, the first and second segments were pressed one into another, it was observed in living aphid. **b** The first and second segment are inserted into abdomen (*i.Is* *+* *IIs*). Externally, only the basal part of the first and distal part of the second segment are visible

A maximum length of labium while feeding on the outside of aphid body is about 2 mm (Fig. 5a, b), and it is almost sixfold reduced compared to the non-feeding phase (Fig. 1a–c).

**Fig. 6:**
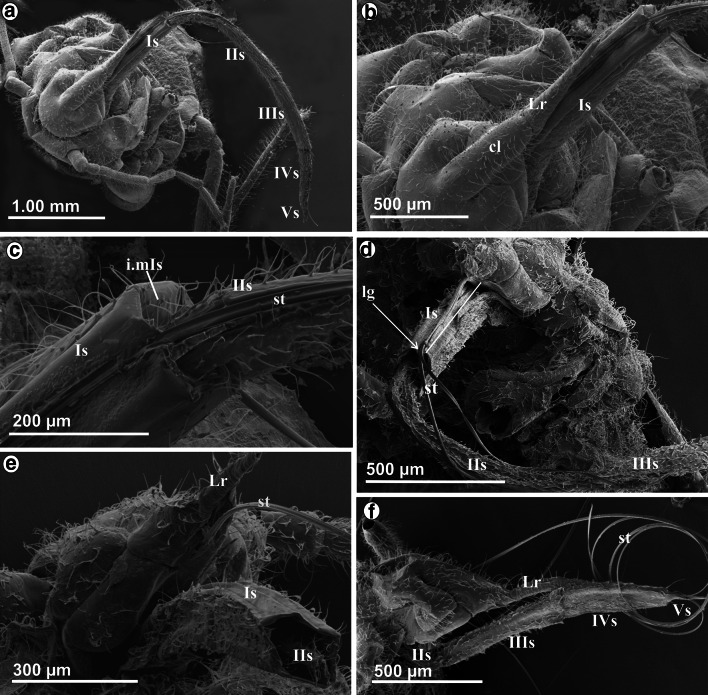
Different phases of shortening of the labium in *Stomaphis quercus*. **a**, **b** The first and second segments evidently shorter. **c** Surface of the first segment was reversed (*i.mIs*) and pulls the second segment. **d** Externally, a portion of the second segment is visible within the first, the stylets are free not placed in the groove. **e** Labrum, stylets, first and second labial segment during the shortening process. **f** *Lr* labrum reaching proximal part of the *IVs* segment the labium in the phase of feeding, stylets are distinctly protruding

**Fig. 7:**
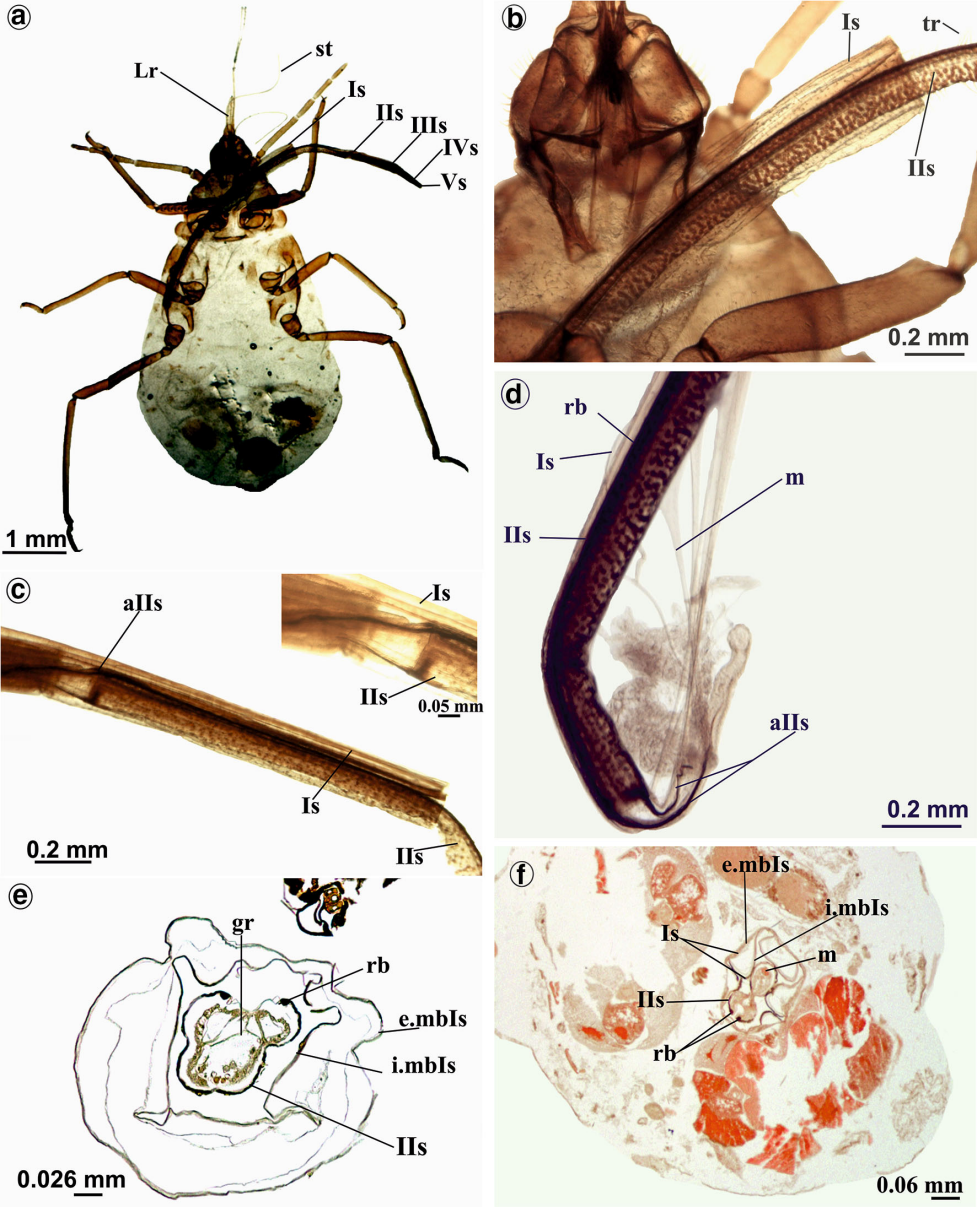
Note how the second segment is rolled in the first in *Stomaphis graffii*. **a**, **b** Shortening of the labium during feeding, part of the first and second segments placed inside the body, externally, a portion of the second segment is visible within the first. **c** Apodema (*aIIs*) of the second segment is placed inside the first segment. **d** A muscles (*m*) and apodema (*aIIs*) of the proximal part of the second segment. **e** Segment II of the labium has been pushed within the first segment forming a sheath (*e.mbIs*, *i.mbIs*) around the second (*IIs*). **f** Same as figure **e**, however, both segments are placed among internal organs in abdomen

Feeding in *S. quercus* and *graffii* is possible when labium is shortened (Figs. 5a, b, 6a, 7a).

*Stomaphis* in the feeding position extends the labium forwards. It probably begins with the *fourth* and *fifth* segments usually being hooked in the bark causing anchoring of the labium. It is possible that after anchoring the labium, the aphid moves back whereas labium uncoils under the body and assumes the horizontal position (Fig. 5a).

At the beginning of feeding, the *first* segment pulls itself inside and is followed by the *second* segment of labium (Figs. 6a–f, 7a–e). The process of labium shortening involves the telescopic insertion of the *second* segment (IIs) into the *first* one (Is) (Figs. 6a–e, 7a–e). In this case, it is the inversion of the distal part of the *first* segment to which the proximal part of the *second* segment is connected. This process is regulated by muscles (m) (Fig. 7d) and a pressure of the body mass of *Stomaphis*. The muscles are attached to the apodeme (aIIs) at the proximal end of IIs (Fig. 7c, d), run along the Is and are attached to the thorax. Their contraction pulls the *second* segment (IIs) into the *first* one (Is) causing its inversion, which starts at the point of their connection (Figs. 6c–e, 7b, c). When the *second* segment is pulled by muscles (Fig. 7d), the *first* membranous one is drawn into the body (Fig. 7f). On the cross section through abdomen, the inversion phase of the *first* segment is visible: the external (e.mbIs) wall and inverted external wall (i.mbIs) (Fig. 7f). Then, the whole *first* segment together with the *second* one (Fig. 7a, b) inverts into the body to the end of abdomen, in the free space between the tissues and organs (Fig. 7f). Both segments move into the body (abdomen), most probably by the pressure of the aphid body mass, as no specialized muscles are localized in abdomen (Figs. 5b, 7f). Probably, the pressure of hemolymph causes compression of the IIs and inverted membrane of Is adheres to it and is significantly folded.

In such a way, the *first* and the *second* segments are hidden inside the body, which causes the exposure of the stylets (st) (Fig. 6f) distinctly protruding from the last segment of the labium. At this phase, the possibility of inserting stylets more deeply into the tissue is increased.

**Fig. 8:**
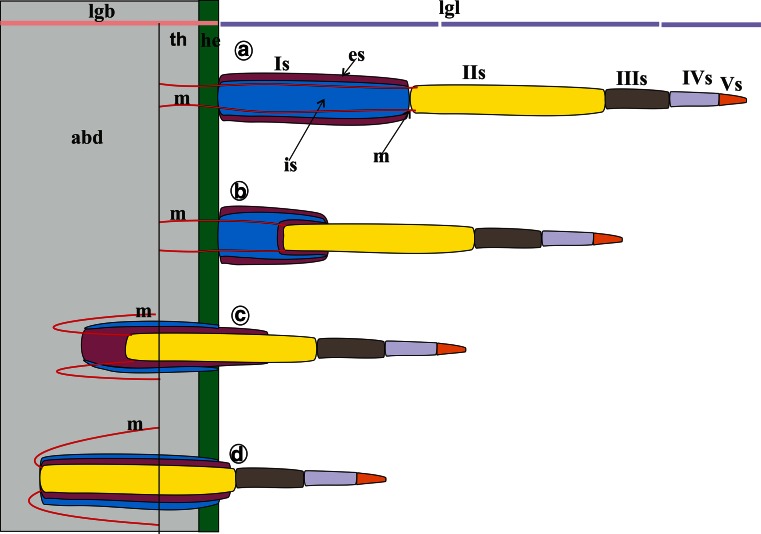
Model of shortening (withdrawing) the first two segments of the labium into abdomen during feeding in *Stomaphis*. **a** All labial segments (*Is–Vs*) protruding externally of head. **b** Inversion of the distal area of the first segment and pushing the proximal part of the second segment. **c** Entire surface of the first segment has been reversed and inside there is the second segment, both are localized in the body (thorax and partly in the abdomen). **d** Total reversal of the first segment and retracting of the second segment, both segments are placed in the abdomen. *abd* abdomen, *es* external surface, *he* head, *is* inverted external surface, *lgb* total body length, *lgl* length of the labium, *m* protractor muscles, *th* thorax, *I–Vs* number segments of the labium

In *Stomaphis*, the process of labium shortening, without the shortening of the stylets, can be presented on the basis of the observation of several stages visible at the individual images (Fig. 8a–d). The most essential stage is the moment when the *second* segment is pulled into the *first* one and then they are placed together in the abdomen (Fig. 8b, c). Externally, the basal part of the *first* segment (Is) as well as the distal part of the *second* one (IIs) are only slightly visible (Figs. 6b, 8d). The *third*, *fourth* and *fifth* segments do not change their shape or length in comparison with the first phase (Fig. 1a, c). In this process, a considerable length of stylets is exposed from the labium.

## Discussion

The present study focused on describing the shape of the labial segments and the stylets bundle. It is assumed that these structures underwent specific evolutionary changes in different taxonomic groups of the aphids in relation to their feeding habits. These changes are manifested as various modifications and forms of particular structures of labium and stylets and provide an interesting material for studies. A clear difference in the structure of the labium in different aphid species seems to testify to such evolutionary changes.

### The modification of labium and stylets

Taxonomic characters of rostrum are mostly confined to the length and shape. Apparently, feeding on tree trunks caused an extensive modification of the mouthpart structure in *Stomaphis* manifested by extremely elongated stylets and rostrum. In tree feeding aphids, this phenomenon has occurred repeatedly since at least the Lower Cretaceous (Heie and Azar 2000; Wegierek and Grimaldi 2010; Homan and Wegierek 2011).

Another example indicating the possibility of elongation of the labial segments and stylets is provided by larvae of the genus *Prociphilus* (Eriosomatinae) which feed on the bark slits of *Abies* roots; their rostrum is significantly longer than the body (Heie 1980). Other aphids, such as *Cinara puerca* Hottes (Hottes 1954) and *Schizoneura lanigera* Hausmann (Davidson 1914), show the same tendency as well.

A specific morphological and functional adaptation can be observed in balsam woolly aphid *Adelges picea* (Ratz.) (Adelgidae), which penetrates the bark of the trees and inserts its extremely long stylets into the wood. Its stylets are about twice the length of the body in adults and four times in larvae; however, the labium is short (Forbes and Mullick 1970).

In several species of Aphididae, e.g., in *Myzus persicae* (Sulzer) (Forbes 1969) and *Aphis fabae* Scopoli (Weber 1928; Hardie et al. 1995), the labium is generally short and consists of four segments. Another type of labium is observed in *Aphis citricola* van der Goot (Razaq et al. 2000), in which the labium is short and three segmented. In representatives of Lachninae, e.g., in the studied genus *Stomaphis* (Pashchenko 1988), the first (basal) segment of labium is almost as long as the body, cylindrical and less sclerotized. Our results indicate that this segment is very characteristic and distinctly different. Its dorsal surface is shaped as a flexible lobe and, because its shape is changeable, this segment seems to play a specific role. Its dorsal side is almost without the trichoid sensilla. The second segment is similar in length to the first one and trough shaped, but it is only retractable preserving its shape.

In most aphids, rostrum is stiff and usually retracted extending backwards between the legs (Foottit and Richards 1993). However, in *Stomaphis*, it has been observed that in natural position, the rostrum is frequently held laterally along the body due to the flexibility of the first segment.

In all previously studied species of aphids (Forbes 1969, 1977; Razaq et al. 2000) as well as in *Stomaphis,* the stylets bundles are externally identical and conform to a common pattern established in Aphididae. No distinct differences were observed in the ending of the mandibular stylets. In several studied species, a few similar small barbs or tooth-like tubercles have been found in this area.

The comparison of stylets bundles in aphids suggests that their length is quite diverse among species, e.g., in *Macrosiphum albifrons* Essig, and the stylets (maxillary and mandibular) are significantly longer (1000 µm) than those in *Therioaphis maculata* (Buckton) (330 µm) (Forbes 1969, 1977) and in *Myzus persicae* (Sulzer), which measures 502 µm for winged and 492 µm for apterae morphs (Forbes 1969), while in *Stomaphis*, they are the longest (about 11,000 µm). Documented stylet lengths range from 0.12 mm (120 µm) in *Rhopalosiphum maidis* (Fitch) (Aphididae) attacking leaf mesophyll (Bing et al. 1991) through 1.5–1.9 mm (1500/1900 µm) in various species of *Adelges* (Adelgidae) consuming cortical parenchyma (Balch 1952; Kloft 1955; Forbes and Mullick 1970) to a known maximum of 12.5 mm (12,500 µm) in *Longistigma caryae* Harris (Aphididae) feeding on stem phloem (Dixon 1975). This indicates that the species sucking stem phloem have the longest stylets. Obvious differences in the labium structure between *Stomaphis* and other aphids are undoubtedly connected with their feeding habits.

### Exceptional adaptation of rostrum to feeding in Stomaphis

Members of *Stomaphis* feed on tree trunks of deciduous trees, so their maxillary and mandibulary stylets must always penetrate deeply into phloem through a thick layer of the cortex. Other aphid species feed mainly on the leaf and stalk, so the stylets get into phloem through a thin layer of epidermis (Bornman and Botha 1973; Dixon 1998; Klingauf 1987). The flexible condition and the same length of the first and second segments of the labium evolved to allow the first segment to be inverted and the second to be inserted into the first one.

While feeding, both segments are shifted to the inside of the body; thus, the labium is shorter and the third, fourth and fifth segments are external. With a shorter labium, partly released styles can penetrate deeply into the plant tissue. When aphids are not feeding, the stylets are kept in the long labium of *Stomaphis*. In larvae and adults of *Adelges picea*, a free long loop of the stylets bundle was frequently observed to extend outward from the rostrum between the labrum and labium. However, the mobile larvae also have stylets bundles as long U-shaped loops in a long crumena (Forbes and Mullick 1970). The crumena in larvae of some aphids, e.g., in *Chermes* was indicated by Heriot (1936). In our study, a loop of stylets rolled in the head and passed to the thorax in the larvae of *Stomaphis graffii*. It was assumed by the previous authors that the stylets in larvae are placed in the crumena—a chitinous wall lined with typical epidermal cells throughout its length and that a special internal pouch is present in larvae of *Stomaphis*. However, Pesson (1951) observed the loop of stylets in the larvae in *Stomaphis* but did not comment on the presence of the crumena.

This structure is also characteristic in other hemipteran insects with very long stylets and is present in sternorryhchan taxa such as Aleyrodidae, Coccidae and Psyllidae (Pollard 1970, 1973; Maranhão 1978, Grimaldi and Engel 2005) and Aradidae (Heteroptera) (Weber 1930; Schuh and Slater 1995).

In psyllids (*Psylla pyricola* Foerster), the fully retracted coiled stylets are under tension and stylet extension generates increasing tension so that when retracted, the stylets readily recoil within the crumena. Penetration of leaf tissues by the stylets bundle depends on the interaction between stylet muscles, opening and closing of the labial clamp, the barbed stylets tips and the ventral position of labium (Ullman and McLean 1986). Similar interactions between stylet muscles may play a role in penetrating plant tissues in *Stomaphis.* However, *Stomaphis* feeds with its labium protruding in an almost horizontal position. It is difficult to understand how sufficient force can be transmitted from the protractor muscles at the base of stylets to the tips of stylets by such a long and slender bundle. Likewise, the mechanism of inversion of labium segments I and II is difficult to explain because only the muscles drawing the II segment into the I were detected. Further insertion of both segments into the body may result from the pressure of body mass or a mechanism connected with the movement of stylets. In case of *Stomaphis*, stylets are not under tension and do not show a tendency to be coiled. There may be some sort of under pressure of hemolymph in the cavity of body resulting in suction of hemolymph from labium and its inversion further inside the body. However, more investigation is necessary to draw any conclusions.

The mechanism of labium shortening in *Stomaphis* during feeding can be regarded as an exceptional adaptation. So far, such phenomena have been observed generally in *Stomaphis* and in several *Cinara species* (Hottes 1954). However, in the latter species, the fifth segmented labium is evidently shorter than that in *Stomaphis*. A report on the possibility of labium shortening in *Stomaphis yanonis* Takahashi was presented by Sorin (1966); however, the process was not explained in any details.

In many phytophagous heteropteran insects having a long labium, the first segment is kept straight and holds the stylets, the second and third segments of rostrum are folded back externally from the bundle, while the fourth segment is situated perpendicularly to the host’s surface and also holds the stylets bundle. However, *Stomaphis* is exceptional in the fact that the labium is kept under of obtuse angle (an almost horizontal position) and the stylets are inserted in this way. Another example is *Diaphorina citri* Kuwayama (Hemiptera: Psyllidae): as soon as it starts feeding, the insect assumes the characteristic feeding position, forming a 45° angle in relation to the leaf surface, favoring the contact of bucal apparatus with the surface (Garzo et al. 2012).

Generally, when feeding on plants, the insect is positioned head down toward the soil and the stylets are inserted perpendicularly into the surface in many aphids (Forbes 1969), planthoppers and leafhoppers (Malone et al. 1999; Leopold et al. 2003). While the insect is not feeding, the mouthparts are directed backward toward the body and the stylets are usually withdrawn into the labium. Thus, the *Stomaphi*s species have developed a specific process of labium shortening in contrast to simple and quick labium shortening mechanisms by bending the second segment in some heteropterans (McGavin 1993) or by folding of the base of the basal segment in auchenorrhynchan cicadellids species (Pollard 1968).

Certainly, in species of the genus *Stomaphis*, the long labium protects long stylets. The frequency of altering feeding sites on the host plant during the life cycle of this aphid is not precisely known so far. This model of labium would be disruptive for frequent changes of feeding location. A recent report by Depa et al. (2014) suggested that the retraction of a labium part from body and the retraction of the stylets from plant tissues may take several minutes. Thus, a rapid escape and refuge from predators seems hardly possible. The shape of the labium may be partly accounted for by a hidden and almost sedentary type of life preferred by *Stomaphis*. The selection of a single location on the plant, adopting a characteristic position while feeding and penetration of phloem for a long time is beneficial for this aphid. In the observation conducted by Depa (2014, unpublished), a single specimen of *Stomaphis longirostris* on *Populus nigra*, attended by ant *Lasius fuliginosus,* continued the feeding process for 60 h at the same location on the tree trunk (unfortunately the place of feeding was destroyed and observation was terminated). Additionally, the species of *Stomaphis* are protected from the predator by mutualism with some species of ants (Depa et al. 2014), so a stable feeding place is possible.
